# Genome Diversity of Epstein-Barr Virus from Multiple Tumor Types and Normal Infection

**DOI:** 10.1128/JVI.03614-14

**Published:** 2015-03-18

**Authors:** Anne L. Palser, Nicholas E. Grayson, Robert E. White, Craig Corton, Samantha Correia, Mohammed M. Ba abdullah, Simon J. Watson, Matthew Cotten, John R. Arrand, Paul G. Murray, Martin J. Allday, Alan B. Rickinson, Lawrence S. Young, Paul J. Farrell, Paul Kellam

**Affiliations:** a Wellcome Trust Sanger Institute, Hinxton, Cambridge, United Kingdom; b Section of Virology, Imperial College Faculty of Medicine, London, United Kingdom; c School of Cancer Sciences, University of Birmingham, Birmingham, United Kingdom; d University of Warwick, University House, Coventry, United Kingdom; e Division of Infection and Immunity, UCL, London, United Kingdom

## Abstract

Epstein-Barr virus (EBV) infects most of the world's population and is causally associated with several human cancers, but little is known about how EBV genetic variation might influence infection or EBV-associated disease. There are currently no published wild-type EBV genome sequences from a healthy individual and very few genomes from EBV-associated diseases. We have sequenced 71 geographically distinct EBV strains from cell lines, multiple types of primary tumor, and blood samples and the first EBV genome from the saliva of a healthy carrier. We show that the established genome map of EBV accurately represents all strains sequenced, but novel deletions are present in a few isolates. We have increased the number of type 2 EBV genomes sequenced from one to 12 and establish that the type 1/type 2 classification is a major feature of EBV genome variation, defined almost exclusively by variation of EBNA2 and EBNA3 genes, but geographic variation is also present. Single nucleotide polymorphism (SNP) density varies substantially across all known open reading frames and is highest in latency-associated genes. Some T-cell epitope sequences in EBNA3 genes show extensive variation across strains, and we identify codons under positive selection, both important considerations for the development of vaccines and T-cell therapy. We also provide new evidence for recombination between strains, which provides a further mechanism for the generation of diversity. Our results provide the first global view of EBV sequence variation and demonstrate an effective method for sequencing large numbers of genomes to further understand the genetics of EBV infection.

**IMPORTANCE** Most people in the world are infected by Epstein-Barr virus (EBV), and it causes several human diseases, which occur at very different rates in different parts of the world and are linked to host immune system variation. Natural variation in EBV DNA sequence may be important for normal infection and for causing disease. Here we used rapid, cost-effective sequencing to determine 71 new EBV sequences from different sample types and locations worldwide. We showed geographic variation in EBV genomes and identified the most variable parts of the genome. We identified protein sequences that seem to have been selected by the host immune system and detected variability in known immune epitopes. This gives the first overview of EBV genome variation, important for designing vaccines and immune therapy for EBV, and provides techniques to investigate relationships between viral sequence variation and EBV-associated diseases.

## INTRODUCTION

Epstein-Barr virus (EBV) infects about 90% of the world's population and plays a role in many human diseases. EBV persists latently in infected B cells for the lifetime of the infected individual, residing as a multicopy episome and replicating with each cell division. EBV causes infectious mononucleosis and is causally associated with several types of cancer, including endemic Burkitt lymphoma (BL), nasopharyngeal carcinoma (NPC), 30% of Hodgkin lymphoma cases, 10% of gastric carcinoma cases, and some cases of diffuse large B-cell lymphoma (DLBCL) and leiomyosarcoma (1). In immunosuppressed individuals, the ability of EBV to cause long-term proliferation of infected B cells results in immunoblastic lymphomas, the main cause of EBV disease in transplant patients (2). In total, EBV is associated with approximately 1.5% of human cancers worldwide. Furthermore, epidemiological evidence points to the involvement of EBV in the autoimmune disorders multiple sclerosis and systemic lupus erythematosus (2).

Some diseases associated with EBV have notably different incidence rates throughout the world; NPC is exceptionally frequent in southern China, and endemic BL is very frequent in sub-Saharan Africa, where malaria is hyperendemic (1). Many factors are likely to contribute to the incidence of EBV-associated diseases in different geographic populations, although the role of EBV sequence variation is not yet well defined. It is known that EBV genome variation can contribute to lymphomagenesis; deletion of the EBNA3B gene enhances EBV tumorigenicity in a mouse model reconstituted with the human immune system from hematopoietic stem cells (3). The tumors arising in those mice resembled DLBCL, and some human DLBCL cases contain EBV with a deletion or disruption of the EBNA3B gene. EBV genome variation has also been observed in Burkitt lymphomas, where approximately 10% of tumors contain EBV with a deletion of EBNA2 and the C-terminal exons of EBNA-LP (4). This suggests that EBV variants may differ in their ability to cause disease, although it is not known if such variants are transmissible between individuals or arise spontaneously within an individual and are not transmissible. Since the establishment of lifelong persistence of EBV might involve the transit of infected B cells through germinal centers (where the enzyme activation-induced cytidine deaminase [AID] promotes DNA mutation), the potential exists for EBV genome variants to arise during long-term infection. To investigate the potential for genome variation in EBV to affect the phenotype of different strains, a more extensive analysis of EBV genomes is required. Efforts to produce successful EBV vaccines (5) will also depend on ensuring that the vaccines are directed against the sequence of currently circulating isolates. Understanding more about EBV sequence variation in normal infection and disease is thus of considerable interest.

There are known differences in phenotypic properties between EBV isolates. B95-8 EBV lacks some of the BART miRNA genes and one of the origins of lytic replication (6) but efficiently establishes lymphoblastoid cell lines (LCLs) from peripheral blood B cells, whereas M81 EBV (derived originally from an NPC) is more efficient at infecting epithelial cells and gives a higher frequency of spontaneous lytic virus replication (7). EBV strains have been classified into type 1 and type 2 (also known as types A and B, respectively) based primarily on the sequence of their EBNA2 gene (1), with the EBNA2 protein sequence of the type 1 reference strain having only 56% identity to type 2 EBNA2. The extent of variation elsewhere in the genome between EBV type 1 and 2 is not well established, as only one type 2 EBV full genome sequence (AG876) has been published (8). Type 1/type 2 variation in the EBNA3 family of genes is generally linked to the EBNA2 variation (9, 10), although intertypic recombinants have been identified (11–13), and some linkage of EBNA-LP variation has also been proposed. Type 1 EBV strains are prevalent worldwide, but in parts of sub-Saharan Africa, type 2 is equally prevalent. The main phenotypic difference is that type 1 EBV transforms human B-lymphocytes into LCLs more efficiently (14). The LMP1 gene has been classified into 7 sequence variants, with some evidence for sequence variation affecting its cell transformation properties (15, 16). Other studies of polymorphisms in EBV genes have been summarized recently (15). However, too few EBV whole-genome sequences have been determined to investigate the range and frequency of disease and geographically associated genome variation.

The first complete EBV genome sequence, B95-8, was published in 1984 (6). Since then, genome sequences of 22 additional EBVs have been reported (AG876, GD1, GD2, HKNPC1, Akata, Mutu, C666-1, M81, Raji, K4123-Mi, and K4413-Mi), as well as eight NPC EBV sequences and three EBV genomes derived from the 1000 Genomes project (7, 8, 17–24). Recently, we and others have described herpesvirus sequencing strategies using a genome capture method analogous to human exome sequencing (18, 25). In addition to the sequencing itself, methods of assembling, annotating, and analyzing the 170-kb EBV genomes have also been established. We have successfully applied these methods to a large set of available EBV isolates, and we describe 71 new EBV genomes from different locations around the world; a combined analysis of these alongside 12 previously published sequences provides the first opportunity to test the general validity of the EBV genetic map and explore recombination, geographic variation, and the major features of variation in this virus.

## MATERIALS AND METHODS

### Virus strains.

DNA samples from cell lines and primary tumor biopsy material for sequencing were provided by Paul Farrell, Martin Allday, and Robert White at Imperial College London, Paul Murray, Alan Rickinson, and John Arrand at the University of Birmingham, and Lawrence Young at the University of Warwick. Sample names, EBNA2 types, and geographic origins are listed in Table 1. The Saliva1 sample (2 ml) was collected from a healthy individual in the United Kingdom using an Oragene saliva kit (Labtech), and DNA was extracted directly from the kit according to the manufacturer's instructions.

**TABLE 1:**
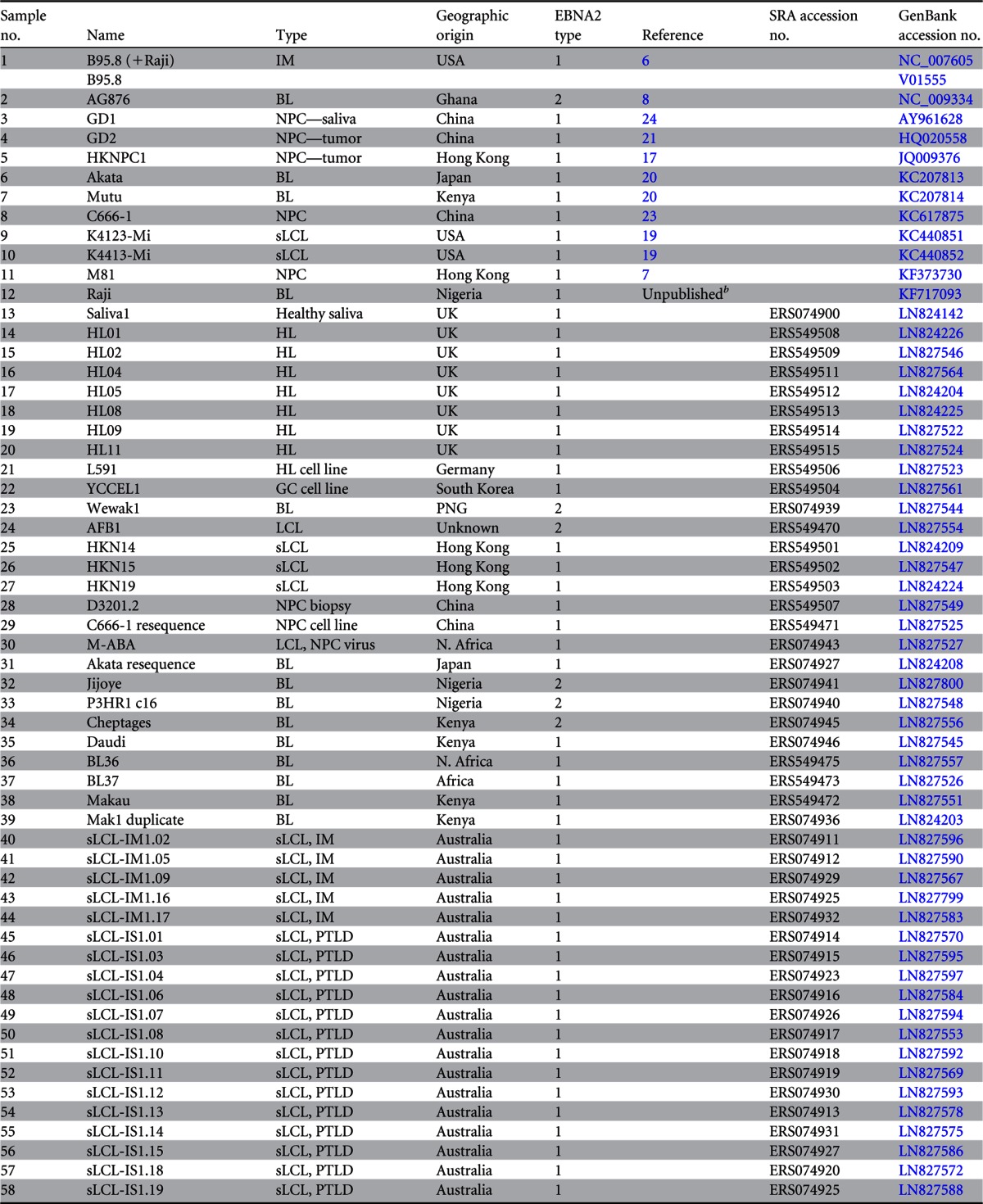

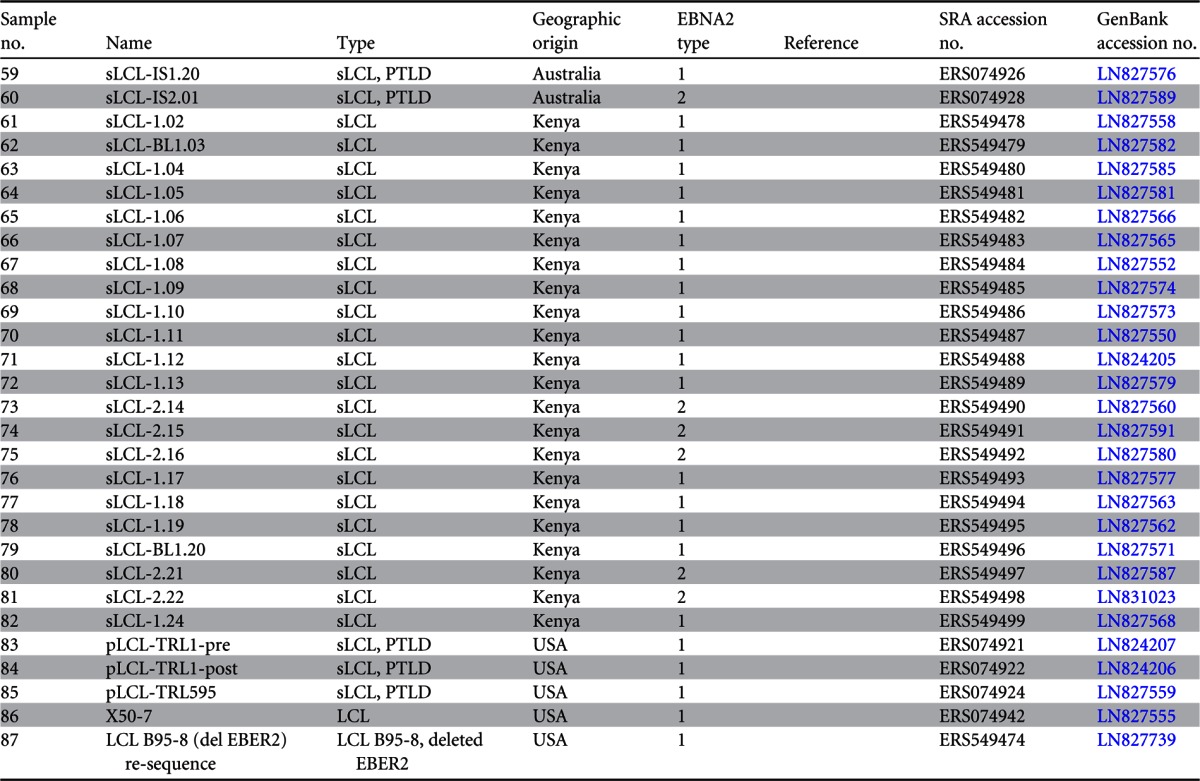
List of samples sequenced and the 12 published sequences analyzed^*a*^

a BL, Burkitt's lymphoma; GC, gastric carcinoma; HL, Hodgkin's lymphoma; IM, infectious mononucleosis; LCL, lymphoblastoid cell line; sLCL, spontaneous lymphoblastoid cell line; NPC, nasopharyngeal carcinoma; PTLD, posttransplant lymphoproliferative disease; PNG, Papua New Guinea; N. Africa, North Africa.

b The Raji sequence was provided by Wolfgang Hammerschmidt prior to publication.

### Virus load.

Virus load was measured by real-time PCR of a 78-bp amplicon spanning the intron between exons W1 and W2 of EBNA-LP within the major internal repeat of EBV. Samples were prepared with the SensiMix dU kit (Bioline) using a 5 mM MgCl_2_ concentration, forward and reverse primers at a 20 pM final concentration (forward primer, 5′ GGCCAGAGGTAAGTGGACTTTAAT 3′; reverse primer, 5′ GGGGACCCTGAGACGGG 3′), and a probe at a 10 pM final concentration (5′ FAM-CCCAACACTCCACCACACCCAGGC-BHQ1 3′). Quantitative PCR was performed on a Masterplex thermocycler ep (Eppendorf) with an initial 15-min incubation at 95°C followed by 45 cycles at 95°C for 15 s and 60°C for 60 s. Threshold cycle (*C_T_*) values were compared to a standard curve generated using a B95-8 plasmid target to assign a copy number per microliter. Samples with more than 10^6^ total genome copies were sequenced.

### Bait design.

Overlapping 120-mer RNA baits spanning the length of the reference genomes (accession numbers NC_007605 and NC_009334 for type 1 and type 2 EBV, respectively) were designed using eArray software (Agilent Technologies). Baits were designed so that each nucleotide was covered by 5 different bait sequences, leading to a total of 7,154 probes covering type 1 EBV and 7,193 covering type 2 EBV with a new bait every 24 bp of the genome. Bait libraries were synthesized by Agilent Technologies.

### Target enrichment and sequencing.

One to three micrograms of each DNA sample was sheared to 200- to 400-bp fragments using a Covaris E210 sonicator (Covaris Inc.). End repair, nontemplated addition of 3′-A, adaptor ligation, hybridization, enrichment PCR, and all postreaction cleanup steps were performed according to the SureSelect Illumina paired-end sequencing library protocol (version 1.1). Samples were multiplexed (6 to 25 samples per lane on an 8-lane flow cell), and cluster generation and sequencing were performed using an Illumina HiSeq 2000 sequencer. Sequencing reads were 76-bp paired-end reads in FASTQ format with per-base Phred quality scores.

### Genome assembly.

All read pairs were subjected to stringent quality control using the QUASR (http://sourceforge.net/projects/quasr) QC pipeline (26) to remove duplicate read pairs and to remove paired reads if either read had a raw median Phred quality score below 32 or were trimmed from the 3′end until the median Phred score was >32. Any reads less than 50 bp in length after trimming were discarded. The remaining quality controlled read pairs were assembled using the VelvetOptimiser (v2.2.4) script (http://www.vicbioinformatics.com/software.velvetoptimiser.shtml), which scans different parameters in the Velvet (v. 1.2.07) *de novo* assembler (27) to optimize the assembly. Contigs generated by Velvet were oriented to the reference genome (NC_007605 for type 1 or NC_009334 for type 2) using Abacas (v1.3.1) (28), and gap filling and contig extension were performed computationally using IMAGE (29) and GapFiller (30). Improved contigs were again oriented to the appropriate reference genome using Abacas. The raw sequencing reads were mapped back to the oriented contigs and checked manually for misassemblies.

### PCR gap filling and verification of *de novo* assemblies.

For genome regions that could not be finished computationally, PCR and capillary sequencing was used. Primer sequences and annealing temperatures used for PCR and sequencing are listed in Table SA1 in the supplemental material. PCR was performed using a Platinum *Taq* DNA polymerase high-fidelity kit (Invitrogen), and cycling conditions were as follows: 95°C for 3 min, 30 cycles of 94°C for 20 s, the specific primer annealing temperature (see Table SA1) for 30 s, and 68°C for 3 min, and a final extension at 68°C for 7 min. PCR with primers U005 and U006 was performed using the KAPA 2G Robust PCR kit with deoxynucleoside triphosphate s (dNTPs) (Kapa Biosystems) using GC-rich buffer and the following cycling conditions: 98°C for 3 min, 25 cycles of 98°C for 30 s, the specific primer annealing temperature for 30 s, and 72°C for 2 min, and a final extension of 72°C for 2 min. PCR products were sequenced on an ABI 3730 capillary sequencer. The same primers were used for sequencing except where noted. PCR products were merged into the *de novo* genome assembly using Gap5 software (31) to generate a final genome sequence for each strain. Regions where PCR failed or which could not be fully resolved were filled with Ns.

### Estimation of IR1 repeat size.

Southern blotting using a probe to IR1 containing BamHI W (positions 13215 to 16287 of NC_007605) was used to estimate by size the copy number of the IR1 repeat in a subset of virus strains. Cell line DNA was digested with KpnI and analyzed by Southern blotting, using a depurination step to aid transfer of the large restriction fragments. The unique region sequences allowed correction for variation in KpnI sites in the EBV genomes. Depth of sequencing was also used to estimate the number of copies of the IR1 repeat, and the results from both methods were compared. The short reads from each sample were mapped to a single copy of the IR1 repeat (3,071 bp; sequence taken from the B95-8 reference genome, NC_007605) using the Burrows-Wheeler aligner (BWA) (32), and the average depth of coverage was calculated using SAMTools (33). Reads were also mapped to a single-copy gene (BALF5) to estimate the genome coverage in nonrepetitive parts of the genome. The average depth of the IR1 mapping was normalized to the average depth of BALF5 to estimate the number of copies of the IR1 repeat in each sequence.

### Genome alignments, SNP calling, and phylogenetics.

A map of the geographic origins of the samples was generated using Tableau software (v8.8.2). Sequence alignments were generated using MAFFT (v7.0) and EMBOSS Stretcher (v6.6) and parsed using Biopython (34). Repeat regions were masked across the whole alignment using the coordinates of the repeat regions in the NC_007605 reference sequence annotation. Single nucleotide polymorphisms (SNPs) between genomes were counted relative to a consensus sequence derived from the multiple-sequence alignment. LMP1 types were assigned manually from a multiple-sequence alignment of LMP1 protein sequences according to the types defined previously (16). Model selection on an LMP1 nucleotide alignment was performed using jModelTest (v2.1.5) (35), and the most appropriate model was a general time-reversible model of nucleotide substitution, with gamma-distributed among-site rate heterogeneity and a category of invariant sites (GTR+G+I). A phylogenetic tree was generated using maximum likelihood implemented in PhyML (v3.0) (36). Tree topology was assessed by bootstrapping analysis using 600 pseudoreplicates, and bootstrap values above 60 are shown on the tree. The tree was rooted on the longest branch.

### Epitope variation and positive selection analysis.

SNP variations within previously identified T-cell epitopes (51) were called from multiple-sequence alignments of the EBNA3A, -3B, and -3C protein sequences. Sequences without an intact open reading frame were excluded, and repeat regions were masked in the alignment. Positive selection analysis was performed using the Datamonkey web server of the HyPhy package (37, 38). Model selection was performed using jModelTest (v2.1.5) (35), and recombination was screened for using GARD and SBP recombination detection programs (39). Recombination was accounted for by using the trees generated by GARD on either side of confirmed breakpoints. Positive selection was detected using the fixed-effects likelihood (FEL; *P* < 0.05), mixed-effects model of evolution (MEME; *P* < 0.05), and fast unconstrained Bayesian approximation (FUBAR; posterior probability > 0.9) methods (40–42), and codons were reported as under selection if they were significant in two or more programs.

### Principal-component analysis and recombination detection.

Principal-component analysis was performed on all SNPs in the genomes using the scikit-learn package of ScientificPython (43). SimPlot (v3.5.1) (44) and RDP4 (v4.35) (45) were used to detect recombination between strains. Similarity plots and bootscan analysis using a window size of 4,000 and increment of 1,000 were generated using manual bootscan in RDP4.

### Data accession numbers.

All sequence data are available in the European Nucleotide Archive (http://www.ebi.ac.uk/ena) under the study accession number ERP001026 and individual sample accession numbers as listed in Table 1. All genome sequences are available in GenBank under the accession numbers listed in Table 1.

## RESULTS

### Target enrichment, sequencing, and *de novo* assembly of EBV genomes.

**FIG 1:**
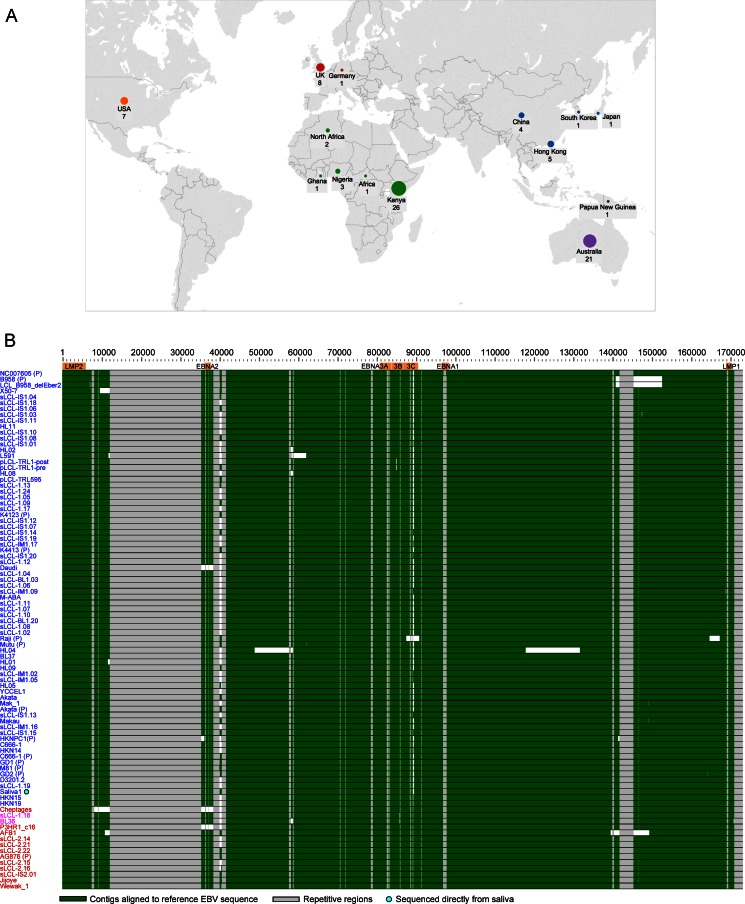
Origin of samples and alignment of new EBV genome sequences. (A) World map depicting the origins of the 83 unique EBV genomes sequenced and analyzed in this study. The sizes of the circles are proportional to the number of samples from each geographic region, with country and number of genomes annotated. One sample without country information is listed as “African,” and one sample of unknown geographic origin is not shown. (B) Whole-genome multiple-sequence alignment of all EBV genomes, including the EBV (B95-8/Raji) reference genome (NC_007605, top) and 12 previously published genomes (annotated with a “P”). Strains are ordered by their similarity to the sequence of the type 1 reference strain (NC_007605), shown at the top. Type 1 genomes are in blue, type 2 genomes are in red, and recombinant (type 1/2) sequences are in purple. Contigs are indicated in green. Repetitive regions are masked out in gray. Gaps and deletions are in white. The wild-type genome derived from the saliva of a healthy individual is annotated with a turquoise dot. The HL04 Hodgkin lymphoma biopsy specimen has an inversion of a section of the genome from position 65000 to 125000. Known deletions in Daudi, P3HR1, pLCL-TRL1, and an LCL generated with a B95-8-based BAC (LCL delEBER2) were detected.

The large (172-kb) EBV genome combined with the low abundance of viral DNA relative to host DNA presents a sequencing challenge. A custom target enrichment library for EBV was designed, comprising a series of 120-nucleotide RNA oligonucleotide baits tiled across the EBV genome (7,154 baits covering type 1 EBV and 7,193 covering type 2 EBV) (25). The EBV baits are biotinylated, allowing selective capture of EBV DNA using streptavidin-coated beads. Starting with 1 to 3 μg of DNA, up to 2,000-fold enrichment of EBV DNA was achieved using this method, with 70 to 85% of reads mapping to the EBV genome after enrichment, compared to about 0.04% of reads without enrichment. Multiplexing up to 25 enriched samples per lane of an Illumina HiSeq sequencer produced sufficient 76-nt paired-end reads from each sample to allow *de novo* assembly of the EBV genome with an average coverage depth of >1,000 reads per nucleotide. A *de novo* EBV genome assembly pipeline was established that does not use a reference sequence, thus avoiding bias that may be introduced by reference-based read mapping. Seventy-one novel EBV genomes and 3 previously sequenced strains were sequenced and assembled (Table 1). The novel EBV sequences were from spontaneous lymphoblastoid cell lines (LCLs) from Australia and Kenya, Burkitt lymphoma cell lines, Hodgkin lymphoma primary biopsy specimens and cell lines, NPC cell lines and biopsy specimens, one gastric cancer cell line, and one EBV strain from the saliva of a healthy individual. Combined with 12 published EBV genome sequences, this resulted in a final data set of 83 unique EBV genomes (Table 1). A summary of the geographic origins of the samples is shown in Fig. 1A.

All genomes were assembled into large contiguous DNA sequences (contigs) separated by the repeat regions, which were not resolvable using short Illumina reads. Five regions required closing and validating by PCR and capillary sequencing, including parts of LMP1 and EBNA2 (PCR primers are listed in Table SA1 in the supplemental material). To assess the reproducibility of the sequencing and analysis pipeline, DNA was extracted from the same cell line on two occasions (Mak1 and Makau), and identical EBV genome sequences were obtained (see Fig. SA1 in the supplemental material). Identical sequences were also obtained from two cell lines established a few weeks apart from the same tumor (pLCL-TRL1-pre and -post), in which a previously described 245-bp deletion in EBNA3B (46) was correctly identified. We also resequenced EBV from the cell lines C666-1 and Akata, whose EBV genomes have recently been published (20, 23), and a bacterial artificial chromosome (BAC) containing the B95-8 genome. Twenty-three, three, and three single nucleotide polymorphisms (SNPs) between our data and the published genomes from C666-1, Akata, and B95-8, respectively, were detected. This contrasts the typical several hundred nucleotide differences between any two type 1 EBV genomes sequenced. The total number of SNPs between each of the EBV genomes can be seen in detail in Fig. SA1 in the supplemental material.

### The EBV genome map is representative of cell line, tumor, and wild-type saliva EBV genomes.

Comparison of EBV contigs to the B95-8/Raji reference sequence (NC_007605) showed that most of the newly sequenced genomes were intact in all regions of the genome (Fig. 1B). We observed the known deletions in a B95-8 derived LCL (LCL B95-8 del EBER2) (47) and in EBV genomes from Daudi and P3HR1. Some EBV strains also had novel deletions relative to the reference strain, including a deletion of approximately 10 kb in the BART region of the AFB1 LCL genome sequence, overlapping but distinct from the deletion present in the B95-8 reference sequence. One isolate from a primary Hodgkin lymphoma (HL04) had an inversion of a large part of the EBV genome corresponding to approximately nucleotides 65000 to 125000 in the reference sequence, with large deletions at each end of the inversion (Fig. 1B). Only 5/83 (6%) of the EBV genomes studied here had such large-scale genome disruption.

**FIG 2:**
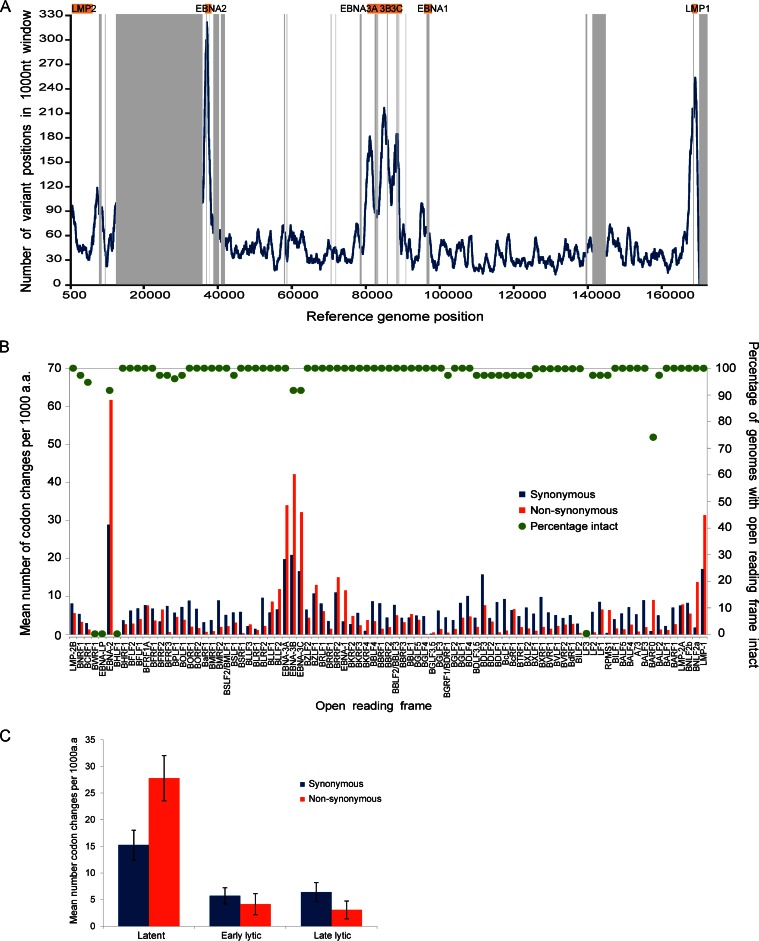
SNP variation across all EBV genomes. (A) Single nucleotide polymorphism (SNP) frequency across 83 unique EBV genomes. The line graph is plotted across the genome showing the number of base positions in a sliding 1,000-nt window where at least one EBV sequence has a SNP relative to the consensus sequence. Repeat regions are masked out in gray. (B) Mean number of codon changes (relative to the consensus of 83 EBV sequences) per gene across the genome, presented as codon changes per 1,000 amino acids to normalize for gene length. Synonymous nucleotide changes are indicated by blue bars and nonsynonymous changes by red bars. The repeat regions within BZLF1, BPLF1, BLLF1, and EBNA1, -2, -3B, and -3C have been masked, and data are provided for the nonrepetitive region only. The right scale shows the percentage of EBV genomes with an intact open reading frame for each gene. BWRF1, EBNA-LP, BHLF1, and LF3 were incompletely assembled due to repetitive regions and were not determined. (C) Numbers of codon changes per gene (means ± standard errors of the means, normalized per kilobase), separated into gene type. Latent genes have an increased number of changes compared to early and late lytic genes. Latency-associated genes also have an increased ratio of nonsynonymous to synonymous coding changes compared to lytic genes.

The multiple-sequence alignment (Fig. 1B) and detailed gene-by-gene analysis of all 83 EBV genomes strongly suggests that the current EBV genome map annotated in NC_007605 is a good representation of the EBV genome. Consistent with this, the open reading frames were shown to be conserved (Fig. 2B). The EBV genome Saliva1 is derived directly from saliva of a healthy carrier and is the first wild-type EBV genome sequenced that was not selected by immortalization of B cells or derived from a cancer cell (Fig. 1B). The close agreement of this sequence to the NC_007605 reference sequence, with no additional insertions or deletions, indicates that the standard EBV genome map is representative of transmissible saliva strains of EBV. The closest EBV strain (based on the fewest SNPs) to the saliva EBV was HKN19, a spontaneous LCL from Hong Kong. Although the identities of the saliva donors tested were anonymous, the panel did include some Asian donors, so it is likely that this is the basis of the similarity.

To estimate the repeat copy number of the major internal repeat (IR1 or BamW repeats), the short reads from each sample were mapped to a single reference copy of the repeat, and the depth of sequence coverage in the repeat array was compared to the depth of coverage in BALF5, a highly conserved unique region of the EBV genome (see Materials and Methods). There was good agreement between the IR1 copy number calculated by this read depth-mapping approach and that from direct measurement of the size of IR1 by restriction enzyme digestion and Southern blotting in a panel of cell lines (see Fig. SA2a in the supplemental material). IR1 varied in copy number from 2 to 9 by sequence mapping, with the most frequent number of internal repeats being 5 or 6 (see Fig. SA2b in the supplemental material). Terminal repeat copy numbers detected in LCL and BL lines are thought to vary considerably; consistent with that, the average terminal repeat copy number determined by Southern blotting in a panel of 17 LCLs or BL cell lines was 7.2, with a standard deviation of 3.7 (data not shown).

### Diversity in EBV genomes is variable across the genome and is highest in latent genes.

To determine which parts of the EBV genome are the most diverse, the number of variant positions within a 1,000-nt sliding window was calculated across all genomes (Fig. 2A). There was substantial variation in SNP frequency across the genome with the highest SNP frequencies correlating with the position of latency-associated genes. The number of synonymous and nonsynonymous codon changes per kilobase of sequence for each EBV gene were also calculated (Fig. 2B), excluding the repeat arrays. The highest number of codon changes were present in the latent genes EBNA2, EBNA3 family, and LMP1. Other genes with high levels of variation include BDLF3 and BLLF1, which encode glycoproteins gp150 and gp350/200, respectively, BZLF1 (immediate early gene), BRRF2 (tegument), and BNLF2a, which is involved in immune evasion. The EBNA3 gene region (including upstream genes BLLF1 and -2), EBNA2, the EBNA1 region (including BRRF2 and BKRF2), and the LMP1 region (including BNLF2a) had a greater ratio of nonsynonymous to synonymous codon changes, suggesting that these genes are evolving under positive selection. Separating genes into latency associated, early lytic, and late lytic showed a clear increase in SNP frequency in latency-associated genes compared to lytic genes and also an increased ratio of nonsynonymous to synonymous codon changes in latency-associated genes (Fig. 2C).

### EBNA3 CD8 T-cell epitope variation.

**FIG 3:**
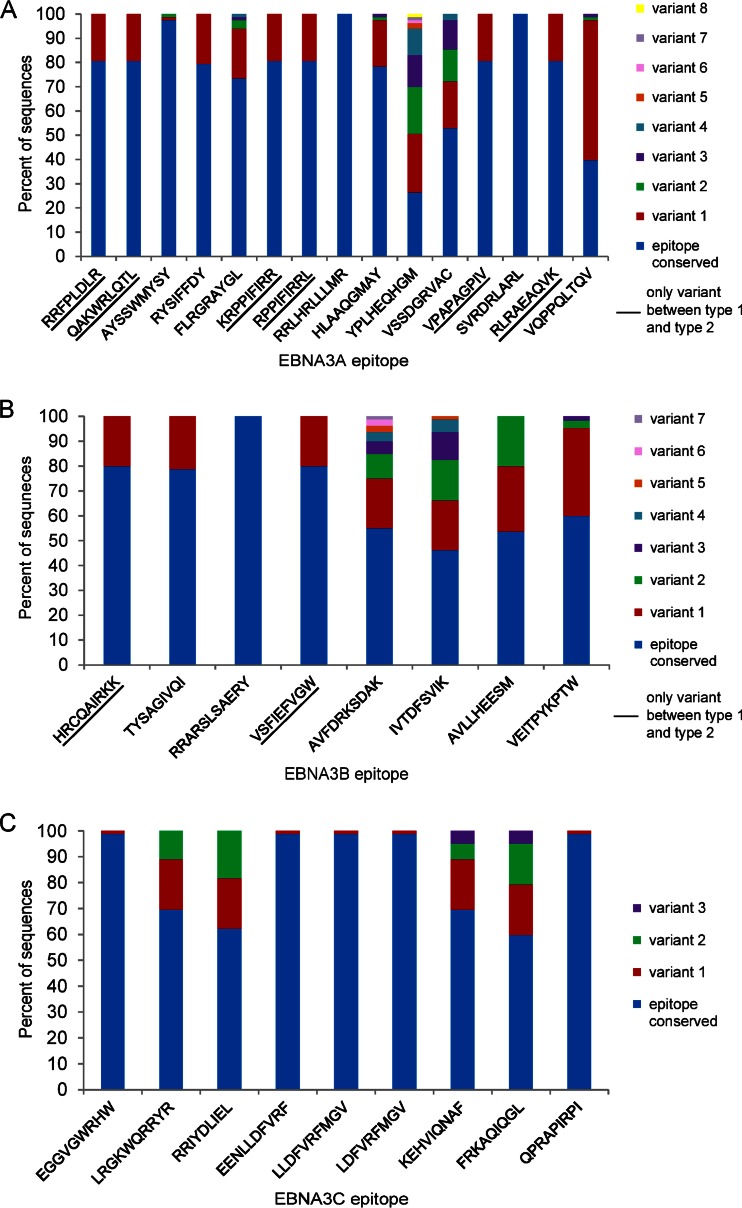
Variation in T-cell epitopes in EBNA3 genes. Variation in known T-cell epitopes in EBNA3A (A), EBNA3B (B) and EBNA3C (C) genes across all 83 EBV genomes. Graphs indicate the percentage of EBV genome sequences with the epitope fully conserved (blue) and the percentage of sequences that have each of the variant sequences as a stacked histogram. Some epitopes are fully conserved across all strains (fully blue bars), some have differences only between type 1 and type 2 (underlined), and some have multiple variants. Only sequences with an intact open reading frame were included.

EBV latency-associated genes (particularly the EBNA3s) are targets of immune recognition during persistent infection, which has been proposed to explain the greater extent of nonsynonymous variation in these genes (48, 51). We therefore examined the known CD8 T-cell epitopes present in the EBNA3 gene cluster (48, 51), listed in Table 2. Two epitopes in EBNA3A (RRLHRLLLMR and SVRDRLARL) were fully conserved across all sequenced genomes (Fig. 3A) and a further six epitopes (RRFPLDLR, QAKWRLQTL, KRPPIFIRR, RPPIFIRRL, VPAPAGPIV, RLRAEAQVK) were variant between type 1 and type 2 sequences but fully conserved within each type (underlined in Fig. 3A). The remaining seven epitopes each had multiple variants present at different frequencies across the data set (Fig. 3A). Similar patterns were seen in EBNA3B (Fig. 3B) and EBNA3C (Fig. 3C), with some epitopes being conserved and some highly variant. In EBNA3B, the immunodominant epitopes AVFDRKSDAK and IVTDFSVIK (12) were the most diverse (Fig. 3B). In general, epitopes were highly conserved within type 2 viruses, with all epitopes being identical to AG876 in all type 2 strains with the exception of EBNA3C epitopes in Wewak1 (one of only two non-African type 2 viruses sequenced). A complete list of all epitope variation is shown in Table 2.

**TABLE 2:** Variation in T-cell epitopes in EBNA3 genes^*a*^

Gene	Epitope	HLA allele	Codons	No. of type 1 sequences with conserved epitope/total	Variation type 1 (no. of sequences)	No. of type 2 sequences with conserved epitope/total	Variation type 2 (no. of sequences)
EBNA3A	RRFPLDLR	B27.05	95–102	67/67		0/16	R1Y (16)
	QAKWRLQTL	B8	158–166	67/67		0/16	QVKWRMTTL (16)
	AYSSWMYSY	A30	176–184	65/67	S3I (1), Y2F (1)	16/16	
	RYSIFFDY	A24	246–253	66/67	R1C (1)	0/16	R1C (16)
	FLRGRAYGL	B8	325–333	61/67	L9I (3), L2F (1), A6V (1), F1L (1)	0/16	F1L,L9Q (16)
	KRPPIFIRR	B27.05	378–386	67/67		0/16	K1R,I7L (16)
	RPPIFIRRL	B7	379–387	67/67		0/16	I6L (16)
	**RRLHRLLLMR**	**B27.05**	**386–395**	**67/67**		**16/16**	
	HLAAQGMAY	ND	450–458	65/67	A3E (1), M7K (1)	0/16	H——– (16)
	YPLHEQHGM	B35	458–466	22/67	P2T (20), H7R (11), H4K (9), P2S (2), M9T (1), P2T (1), P2R (1)	0/16	-PLHQQHSM (16)
	VSSDGRVAC	A29	491–499	44/67	R6Q (11), D4E (10), S2F (2)	0/16	VPKDGRGAC (16)
	VPAPAGPIV	B7	502–510	67/67		0/16	P4L (16)
	**SVRDRLARL**	**A2**	**596–604**	**67/67**		**16/16**	
	RLRAEAQVK	A3	603–611	67/67		0/16	V8A,K9R (16)
	VQPPQLTQV	B46	617–625	33/67	P4T (32), L6V(1), V1E,Q2K (1)	0/16	P4T (16)
EBNA3B	HRCQAIRKK	B27.05	154–157	64/64		0/16	K8Q (16)
	TYSAGIVQI	A24	217–225	63/64	I9L (1)	0/16	I9L (16)
	**RRARSLSAERY**	**B27.02**	**244**–**254**	**64/64**		**16/16**	
	VSFIEFVGW	B58	279–287	64/64		0/16	S2A,I4V (16)
	AVFDRKSDAK	A11	399–408	44/64	A1S,V2L (8), D4N (4), A1S (3), A1P,V2L (2), A1S,V2F (2), A1P (1)	0/16	SVFYRKPDTK (16)
	IVTDFSVIK	A11	416–424	37/64	K9N (13), V2L (9), K9R (1), V2L,K9R (4),	0/16	F5L,V7I (16)
	AVLLHEESM	B35	488–496	43/64	A1T (21),	0/16	RVILHGPPT (16)
	VEITPYKPTW	B44	657–666	39/64	I3L (23), Y6D,K7E (2),	ND, gap	AG876 has VEPTFYQSTW
EBNA3C	EGGVGWRHW	B44	163–171	65/66	R7- (1)	16/16	
	LRGKWQRRYR	B27.05	249–258	57/66	R7K (9)	0/16	Y9F (16)
	RRIYDLIEL	B27.0204.05	258–266	51/66	R2K (15)	0/16	Y4F (16)
	EENLLDFVRF	B44	281–290	66/66		15/16	R9H (1)
	LLDFVRFMGV	A2	284–293	66/66		15/16	V6H (1)
	LDFVRFMGV	B37	286–293	66/66		15/16	R5H (1)
	KEHVIQNAF	B44	335–343	57/66	E2D (5), H3Q (4)	0/16	H3Q,N7K (16)
	FRKAQIQGL	B27.05	343–351	49/66	I6L (13), I6 M (4)	0/16	R2L,I6R (16)
	QPRAPIRPI	B7	881–889	65/66	R3P (1)	16/16	

a ND, not determined. Bold type indicates conservation in all samples.

Positive-selection analysis on the three EBNA3 genes detected several codons under positive selection in each gene. The codons of the EBNA3 coding regions found to be under positive selection (Table 3) were nearly all located outside the established CD8 T-cell epitopes listed in Table 2. Only 1 of 4 codons in EBNA3A, 3 of 12 in EBNA3B, and 0 of 3 in EBNA3C were within known T-cell epitopes. This observation may suggest additional T-cell epitopes yet to be discovered (perhaps related to major histocompatibility complex [MHC] types that have not been studied yet), or these codons might be under positive selection for other reasons, perhaps related to the function of the EBNA3 genes.

**TABLE 3:** EBNA3 amino acids under positive selection

Gene	Codon	No. of methods^*a*^	Amino acid(s)		Location within known epitope
			Reference	Alternate	
EBNA3A	54	2	H	Q, P	No
	459	3	P	T, S, R	458–466, YPLHEQHGM
	681	3	V	A, M	No
	814	3	G	V, A, D, T	No
EBNA3B	33	2	T	Q, K	No
	212	3	T	M, L, R	No
	417	2	V	L	416–424, IVTDFSVIK
	424	2	K	N, R	416–424, IVTDFSVIK
	488	3	A	T, R	488–496, AVLLHEESM
	553	3	P	L, H	No
	614	3	R	W, Q, L, P	No
	738	2	R	Q	No
	847	3	A	E	No
	887	2	A	P, G, V	No
	899	3	G	S	No
	900	3	Q	K, R	No
EBNA3C	104	3	T	A, P	No
	215	2	A	G, E	No
	357	2	G	V, I	No

a Positive-selection detection methods used were FEL, MEME, and Fubar using the HyPhy package.

### LMP1 variation.

**FIG 4:**
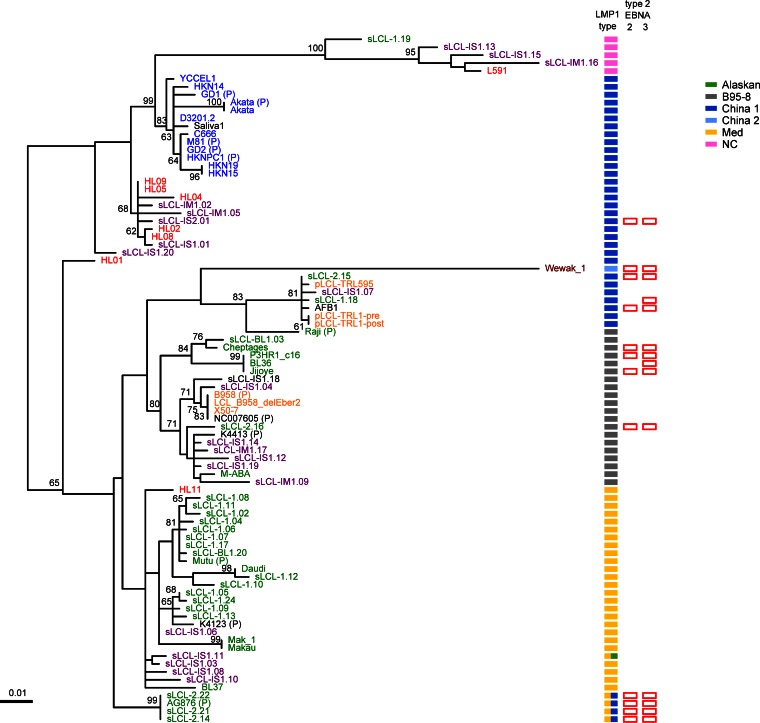
LMP1 phylogenetic tree classified by LMP1 type. Maximum-likelihood phylogenetic tree of LMP1 nucleotide sequence from 83 EBV strains (71 new strains and 12 published strains, annotated with a “P”). Bootstrap values above 60 are shown, and the scale bar represents 0.01 nucleotide substitution per site. All strains could be classified into one of the 7 LMP1 types (as defined in reference 16; see the key for colors), and these define the major clades of the tree, except China 1 strains, which are split across several clades. Strains with type 2 EBNA2 and EBNA3 (empty red bars) are present in multiple LMP1 types but absent from the NC and Med LMP1 types. Geographic origin (shown by the color of the sample name: blue, Asia; green, Africa; red, Europe; yellow, USA; purple, Australia; dark red, Papua New Guinea) shows that many of the LMP1 types, including Med and NC, are found in strains from a wide geographic area.

Variation in the EBV latency-associated gene LMP1 has been described in detail. Immortalized B-cell LCLs, B cells in posttransplant lymphoproliferative disease, and the Reed Sternberg cells of EBV-associated Hodgkin lymphoma all express LMP1 at a high level. LMP1 is also expressed at a low level in NPC and gastric cancers (49). Focusing mainly on its relevance to NPC in China and some other parts of the world, LMP1 variants have previously been classified into seven groups named NC (North Carolina), Med, Alaskan, B95-8, China 1, China 2, and China 3, based on the geographic origins of the original samples (16). A phylogenetic tree of the LMP1 nucleotide sequences from the new EBV sequences determined in this study and previously published genome sequences shows that 6 of the 7 LMP1 types are present, with no observation of the China 3 group. Five genomes have a combination of two LMP1 types (Fig. 4). There is a good correspondence between the LMP1 groups and the LMP1 phylogenetic tree of this larger collection of EBV genomes, with the exception of the China 1 group, which separates into several clades. We also mapped type 2 EBNA2 or EBNA3 onto this analysis. Overall, there is little correlation of LMP1 subtype with the type 1/2 designation of the EBNAs, but it is noticeable that no type 2 EBNA genes are present in viruses with the NC or Med forms of LMP1, even though many of these genomes were from sub-Saharan Africa, where type 2 EBV is most prevalent. It is clear that the NC and Med forms of LMP1 can come from a wide range of geographic regions, suggesting an exclusion of type 2 EBNA2 or EBNA3 in the genomes with NC or Med LMP1. This should be investigated further in larger and more geographically diverse sample sets.

### Type 1 and type 2 classification is a major feature of EBV diversity and is defined by EBNA2 and EBNA3s.

**FIG 5:**
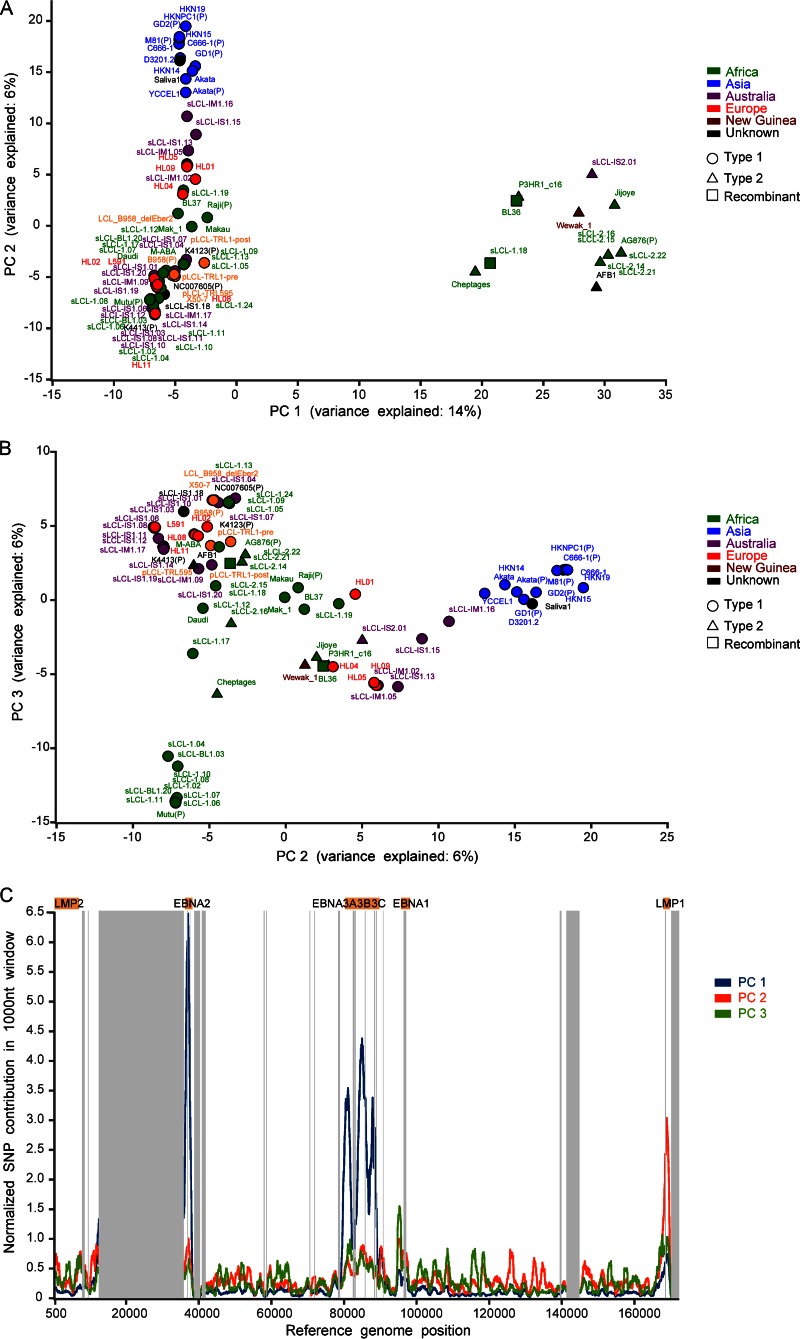
Principal-component analysis separates strains by type 1/2 and geographic origin. Principal-component analysis (PCA) of all EBV strains (71 new strains and 12 published strains, annotated with a “P”) based on SNPs relative to the consensus sequence in a full-genome multiple-sequence alignment. (A) Principal component 1 separates all strains based on type 1 and type 2, with all type 2 strains (triangles) clustering together (including two intertypic recombinants, BL36 and sLCL-1.18, which have type 2 EBNA3s). (B) Principal component 2 shows some geographic clustering of EBV strains, with clear separation of the Asian strains (blue). (C) Regions of the EBV genome that contribute to the first three principal components.

To identify the major features of variation throughout the genome, we performed principal-component analysis (PCA) on SNPs in the genomes. Principal component 1 (PC1) separated EBV strains clearly into type 1 and type 2 (Fig. 5A) and was the single greatest contributor (14% of the variance) to separating the strains. Principal components 2 and 3 provided further discrimination (6% and 6%, respectively) between strains, explained in part by variation associated with geographic origin of the samples (Fig. 5B; strain names are colored by geographic origin). Strains from Asia (blue) clustered separately from other geographic locations, and some other clusters were entirely of African origin. The parts of the EBV genome that contribute to the first three principal components are shown in Fig. 5C. As expected, EBNA2 and EBNA3s account for a large part of PC1 (type 1/type 2), but several genome regions, including LMP1, distinguish PC2. PC3 is more evenly distributed across the genome.

To test the contribution of EBNA2 to the type 1/type 2 designation, we removed the EBNA2 sequence from the genome alignment for all strains and reexamined the PCA. The dominant principal component still showed clustering of strains based on type 1 and type 2 (see Fig. SA3a in the supplemental material), but removing both EBNA2 and the EBNA3 genes eliminated the type 1 and type 2 clustering as the major feature of variation (see Fig. SA3b in the supplemental material). This shows that type 1/type 2 classification, the major feature of variation in EBV genomes, is almost entirely dependent on EBNA2 and the EBNA3s. Further analysis of PC2 and PC3 (see Fig. SA3c in the supplemental material; colored by LMP1 type) shows that, although LMP1 variation is a significant contributor to PC2 (Fig. 5C), strains do not clearly separate by LMP1 type, and other points of variation in PC2 along the genome also play a role. These genome-scale views of variation show that the saliva-derived virus genome clusters with the EBV type 1/Asian/China 1 LMP1 groups, further indicating the geographic origin of this virus.

### Recombination between EBV strains.

**FIG 6:**
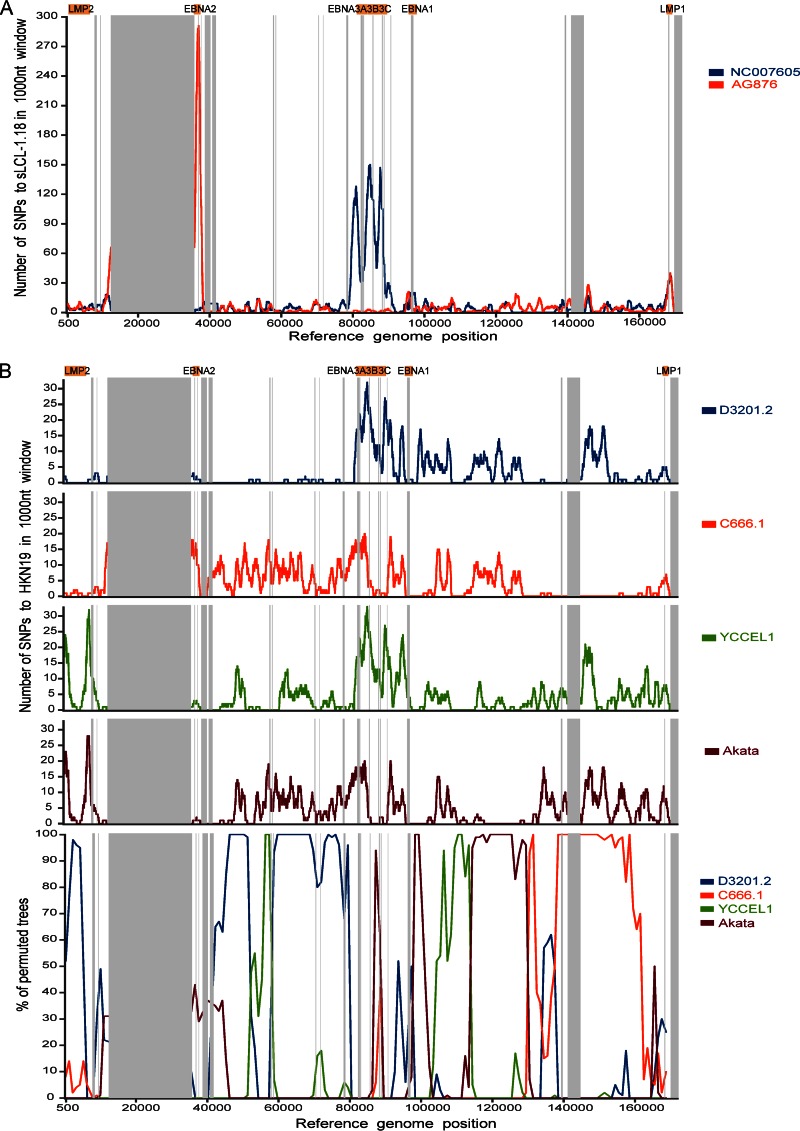
Recombination between different EBV strains. (A) Intertypic recombination between type 1 and type 2 EBV strains. Diversity plots show the number of SNPs per 1,000-nt sliding window comparing EBV strain sLCL-1.18 with the type 1 reference genome (NC_007605; blue) and the type 2 reference genome (AG876; red). Major repeat regions are masked out (vertical gray bars). The sLCL-1.18 EBV genome is highly similar to the type 1 genome in the EBNA2 region (around positions 36000 to 37000) and highly similar to type 2 in the EBNA3s region (around positions 80000 to 89000). (B) Evidence for recombination between different type 1 EBV strains. Diversity and bootscan plots comparing strain HKN19 with 4 geographically related (southeast Asian) EBV strains, with repeat regions masked out (gray bars). Diversity plots (top; number of SNPs in a 1,000-nt sliding window) show high levels of similarity (few SNPs) between HKN19 and strain D3201.2 until approximately position 80000 and similarity to other strains in other parts of the genome. Bootscan plots (bottom) of the same strains confirm that clustering of the strains changes across the genome, indicating the presence of multiple recombination events throughout the genomes.

The established type 1 and type 2 EBV sequence types are normally defined by the EBNA2 sequence, but a small number of intertypic recombinants have been reported (11, 13). Two of the 83 strains analyzed here appear to be intertypic recombinants. BL36, an African Burkitt lymphoma cell line and sLCL 1.18, a spontaneous LCL from Kenya, encoded type 1 EBNA2 and type 2 EBNA3 sequences. Diversity plots generated across the genome between sLCL 1.18 and the type 1 (NC_007605) and type 2 (AG876, NC_009334) reference sequences revealed that in the region corresponding to EBNA2 (around positions 36000 to 37000) there are few SNPs relative to B95-8 but a large peak in the SNPs relative to AG876, indicating a type 1 EBNA2 (Fig. 6A). Conversely, there was a high degree of similarity to AG876 in the region corresponding to the EBNA3 genes (around positions 80000 to 89000) and a peak of SNPs relative to B95-8, indicating type 2 EBNA3 genes. No virus strains with type 2 EBNA2 and type 1 EBNA3s were detected in this set of genomes, consistent with previous studies (11, 13), although this may constitute a sampling bias, as type 1 EBNA2 is more efficient for B-cell transformation *in vitro*.

Extensive recombination in herpes simplex virus 1 was recently described (50). The 83 EBV genomes were therefore examined for the presence of intratypic recombination using RDP4 and bootscanning analysis. Evidence for recombination appeared to be extensive, so analysis was focused on geographically constrained genomes as defined by PCA (Fig. 5B), for example, type 1 EBV sequences from Asia (Fig. 6B). A diversity plot of the number of SNPs between strain HKN19 and 4 other strains from the same geographic region shows that the genome of HKN19 up to approximately position 80000 is very similar to that of EBV strain D3201.2 and from approximately position 130000 is very similar to that of EBV strain C666-1 (Fig. 6B, top). Other regions of the genome show high similarity to strains YCCEL1 and Akata. Bootscan analysis supported this conclusion (Fig. 6B, bottom) and provided evidence for multiple recombination events between EBV genomes. The existence of such recombination should be considered in any phylogenetic analysis of EBV sequence data. Much greater sampling depth, with a more comprehensive and strategic geographical sampling, would be needed to examine the full extent of recombination in EBV genomes worldwide and the contribution this makes to EBV diversity and disease associations.

## DISCUSSION

Genetic analysis of whole virus genomes is essential to understand virus transmission patterns, virus phenotypes, and disease-associated genotypes, although this has not been used extensively in large DNA viruses. Here, we report the sequencing of 71 new EBV genomes, including the first EBV genome sequenced directly from saliva, and analyzed these in combination with 12 previously published strains. This analysis revealed that the established gene map of the EBV genome (NC_007605) is representative of EBV isolates from different parts of the world and from different types of infection. The well-established type 1/type 2 classification was reexamined with the 83 EBV genomes analyzed here and remains the major form of variation, mostly accounted for by variation in EBNA2 and EBNA3A, -B, and -C. In these 83 genomes, there was type-specific linkage between type 2 EBNA2 and type 2 EBNA3 genes (12 strains) and type 1 EBNA2 and type 1 EBNA3 genes (69 strains). There were 2 cases of intertypic recombinants, both with type 1 EBNA2 but type 2 EBNA3 genes. Since most EBV genomes sequenced here are from B cells and type 1 EBNA2 is associated with more efficient B-cell transformation, the overall incidence of intertypic recombinants may be higher due to the possibility of undetected EBNA2 type 2 and EBNA3 type 1 recombinants.

Whole-genome recombination analysis was performed on samples from distinct geographic regions only. We observed intratypic recombination between samples from the same geographic region with breakpoints present throughout the genome, consistent with examples of intratypic recombinants described in EBNA3 genes (3, 51) and a genome-wide study of 20 HSV strains (50). The extent of recombination and the extreme differences in SNP density between the latency-associated and structural genes make phylogenetic trees of large regions of the genome difficult to interpret and prevent the accurate identification of the ancestry of strains at this stage. Such recombination in alpha and gamma human herpesviruses suggests that reinfection (and indeed coinfection of single cells) must occur and that adaptive immunity is not sufficient to prevent this. Whether this will affect herpesvirus vaccine efficacy or provide an environment for vaccine and wild-type herpesvirus recombination should be investigated. As with RNA viruses, it is clear that much larger studies encompassing EBV genome sequences from different locations worldwide and from different disease associations will be required to identify structural variants or mutations that may be associated with EBV disease phenotypes.

A limitation of the target enrichment approach used here is that the EBV bait design used the sequence of the B95-8 and AG876 reference genomes. Thus, any novel sequence present in an isolate might not be captured by the sequencing process. However, there is no specific evidence for this at this stage. If such novel sequences were indeed present, they would appear as unclosable gaps between contigs, but in fact, the only such gaps observed could be accounted for by the presence of repeat regions. As with all short-read sequencing, we did not obtain a unique assembly across the repeat arrays present in the EBV genome; therefore, the genomes are in effect large contigs of assembled sequence separated by the large repeat regions. It should be noted that in previous studies of single EBV genome sequences, presented as complete genomes, a suitable number of identical copies of the repeat elements were inserted to provide a continuous sequence without evidence of the number of copies or that all copies are identical. We have chosen to leave such regions as unassigned, but we have shown that correlating variation in read depth from a repeat region relative to a unique region does provide an accurate way to estimate the repeat copy number for the EBV major internal repeat (IR1). A detailed analysis of IR1 repeat variation will be reported separately.

There is clear evidence for a higher frequency of SNPs in latency-associated genes and also a higher ratio of nonsynonymous changes. This may be consistent with positive selection in these genes, perhaps in relation to MHC types, as has been proposed previously (12, 48). However, most of the codons in EBNA3 genes that were under positive selection were not in known cytotoxic-T-lymphocyte (CTL) epitopes, suggesting either that many more epitopes remain to be discovered or that selection is on other functional features of the EBNA3 proteins (52). There was similar evidence for a higher frequency of nonsynonymous changes in glycoproteins, including gp350 and gp25, which have both been shown to be CTL targets, and the immune evasion gene BNLF2a (Fig. 2B). It is notable that there is a clustering of genes with a high ratio of nonsynonymous changes in certain genome regions, but the reason for this is unclear. The further definition of CTL epitope diversity will have an important impact on CTL immunotherapies that are being assessed to treat an increasing range of EBV-associated malignancies.

In addition to its importance for design of an EBV vaccine, describing the sequence variation of the EBV genome is essential for identifying the role of variation in diseases associated with EBV, particularly diseases whose incidence varies in different geographic locations. For example, for NPC in Southeast Asia, genetic variation of the host, local environmental cofactors (including coinfections), and natural variation in EBV are likely to be important. Our results show that there are many points of variation that distinguish Asian strains of EBV from EBV from other parts of the world; indeed, PC2 in our principal-component analysis corresponds to some extent with the Asian EBV strains. It may be that the endemic strain of EBV in Southern China is inherently more able to contribute to NPC, and it is still possible that a specific EBV variant is involved in that cancer. The recent demonstration (53) of the importance of a single amino acid in EBNA2 for determining the superior growth maintenance function of type 1 EBNA2 shows how very small sequence differences can have a large effect on phenotype in EBV. Our results provide the first global view of EBV sequence variation at the whole-genome level, validate the currently used genetic map, demonstrate an effective method for sequencing large numbers of virus genomes, and create a framework for much larger future studies directed to these goals.

## Supplementary Material

Supplemental material
