# Supplementary material for: Genome Diversity of Epstein-Barr Virus from Multiple Tumor Types and Normal Infection
Source: J Virol. 2015 Mar 18;89(10):5222–37. doi: 10.1128/JVI.03614-14 (PMC4442510; doi:10.1128/JVI.03614-14)
Supplement: Supplemental material [file supp_89_10_5222__index.html]

Genome Diversity of Epstein-Barr Virus from Multiple Tumor Types and Normal Infection — Supplemental material 

# Genome Diversity of Epstein-Barr Virus from Multiple Tumor Types and Normal Infection

## Supplemental material

**Files in this Data Supplement:**

- Supplemental file 1 -

  Fig. SA1 (SNP differences among all available EBV genome sequences.)

  Fig. SA2 (Correlation of sequence depth and repeat array size by Southern blotting in major internal repeat.)

  Fig. SA3 (EBNA2 and EBNA3 contribute to separation of strains by principal components.)

  Table SA1 (List of PCR primers used.)

  PDF, 531K
