# Supplementary material for: Genome Diversity of Epstein-Barr Virus from Multiple Tumor Types and Normal Infection
Source: J Virol. 2015 Mar 18;89(10):5222–37. doi: 10.1128/JVI.03614-14 (PMC4442510; doi:10.1128/JVI.03614-14)
Supplement: Supplemental material [file JVI.03614-14_zjv999090397so1.pdf]

APPENDIX

FIGURE LEGENDS

Figure A1 SNP differences between all available EBV genome sequences

Matrix of SNPs between each genome analysed in this study and each of the other EBV genomes. Previously published strains are annotated with (P). Numbers represent the total number of base positions different between each pair of genomes (SNPs only, indels are excluded and repeat regions are masked). Strains are ordered by their similarity to the type 1 reference strain NC_007605 at the top. Type 1 genomes are in blue text, type 2 genomes in red text and recombinant (type 1/2) genomes in purple text. The number of SNPs are coloured on a sliding scale from dark blue (very similar) to dark green (very different). Type 2 strains group together at the bottom, being least similar to NC_007605.

Figure A2 Correlation of sequence depth and repeat array size by Southern blotting in major internal repeat

(A) The number of copies of the internal repeat (IR1 or W repeat) was estimated by Southern blotting of 16 strains using a probe to IR1 and also computationally by mapping to a single copy of IR1 to calculate the read depth across this region and normalising to BALF5, a single copy gene. There was a good correlation between the two measurements (R^2 value of 0.59).

(B) All EBV genomes sequenced in this study were mapped to a single copy of IR1 to estimate the number of copies of IR1 in each strain (normalised to BALF5). Frequency plot of the

estimated repeat copy number shows that 5 or 6 copies of the repeat are most frequently found in EBV strains.

Figure A3 EBNA2 and EBNA3 contribute to separation of strains by principal components

Principal component analysis (PCA) of all EBV strains (71 new strains and 12 published strains, annotated with (P)) based on SNPs in a full genome multiple sequence alignment.

(A) PCA analysis with EBNA2 masked from the alignment. Type 1/type 2 clustering is preserved.

(B) Masking both EBNA2 and EBNA3 sequences eliminates the separation of strains into type 1 and type 2 in either component, indicating that these genes account mainly for the type 1/type 2 difference. (C) PC2 coloured by LMP1 type. Although LMP1 is a contributor to PC2 (see Figure 6c) strains do not clearly separate by LMP1 type and other points of variation in PC2 also play a role.

TABLES

Table A1: List of PCR primers used

	Start	End	For primer	Forward sequence	Rev primer	Reverse sequence	Anneal temp.
1	36186	36754	U019	CGCTTGTGTTTTGCTTTATC	U020	AAAATGGTGGGTGCTGTC	58
2	57391	58427	U005	GTGTCGTGGCTGATGGTG	U006	CGAGCTTGTGTCCAGTGATG	61
3	77530	78774	U007	GCCTTTCCACCTGTTGAGG	U008	GCAAATGGACTCTCACCTCG	60
4	77708	78451	U023	GCGAACTGGTGGACACATGA	U024	ATGTCACCAGCCCAACACC	60
5	87370	88673	U025	TCATGTTTGTGAGCCGTG	U026	GATCCTCATACCGGGGTT	55
6	88300	88780	U09A	AAGAACAACCCCGGTATGAG	U10A	AGATCCATCCCACTGAGG	55
7	167503	168284	U021	AGGTGTCTGCCAATTCTCGC	U022	TGAACACCACCACGATGACTC	60

Figure A1

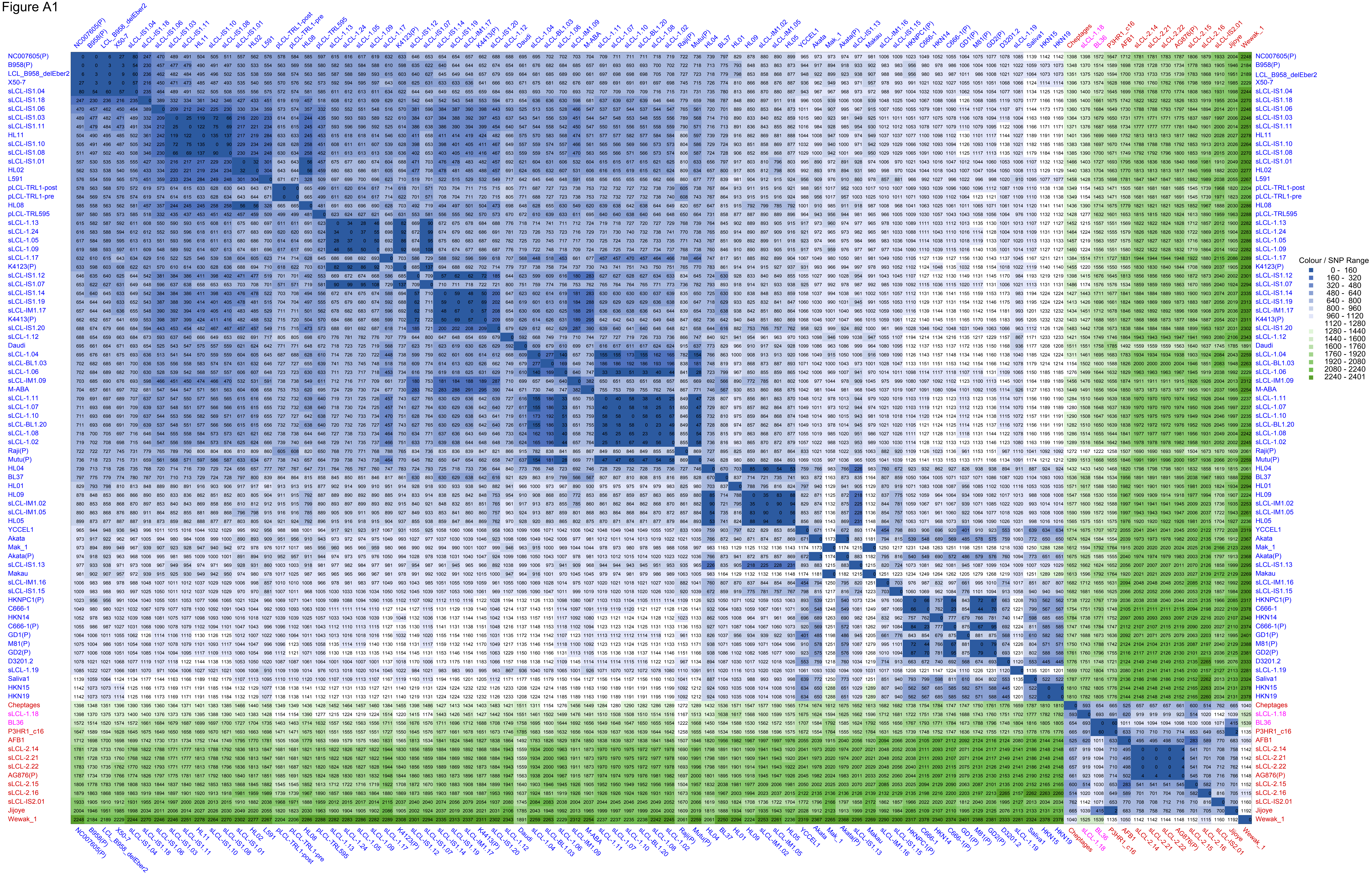

NC007605(P)
B958(P)
LCL_B958_delEber2
X50-7
sLCL-IS1.04
sLCL-IS1.18
sLCL-IS1.06
sLCL-IS1.03
sLCL-IS1.11
HL11
sLCL-IS1.10
sLCL-IS1.08
sLCL-IS1.01
HL02
L591
pLCL-TRL1-post
pLCL-TRL1-pre
HL08
pLCL-TRL595
sLCL-1.13
sLCL-1.24
sLCL-1.05
sLCL-1.09
sLCL-1.17
K4123(P)
sLCL-IS1.12
sLCL-IS1.07
sLCL-IS1.14
sLCL-IS1.19
sLCL-IM1.17
K4413(P)
sLCL-IS1.20
sLCL-1.12
Daudi
sLCL-1.04
sLCL-BL1.03
sLCL-1.06
sLCL-IM1.09
M-ABA
sLCL-1.11
sLCL-1.07
sLCL-1.10
sLCL-BL1.20
sLCL-1.08
sLCL-1.02
Raji(P)
Mutu(P)
HL04
BL37
HL01
HL09
sLCL-IM1.02
sLCL-IM1.05
HL05
YCCEL1
Akata
Mak_1
Akata(P)
sLCL-IS1.13
Makau
sLCL-IM1.16
sLCL-IS1.15
HKNPC1(P)
C666-1
HKN14
C666-1(P)
GD1(P)
M81(P)
GD2(P)
D3201.2
sLCL-1.19
Saliva1
HKN15
HKN19
Cheptages
sLCL-1.18
BL36
P3HR1_c16
AFB1
sLCL-2.14
sLCL-2.21
sLCL-2.22
AG876(P)
sLCL-2.15
sLCL-2.16
sLCL-IS2.01
Jijoye
Wewak_1
Colour / SNP Range
0 - 160
160 - 320
320 - 480
480 - 640
640 - 800
800 - 960
960 - 1120
1120 - 1280
1280 - 1440
1440 - 1600
1600 - 1760
1760 - 1920
1920 - 2080
2080 - 2240
2240 - 2401

Figure A2

A

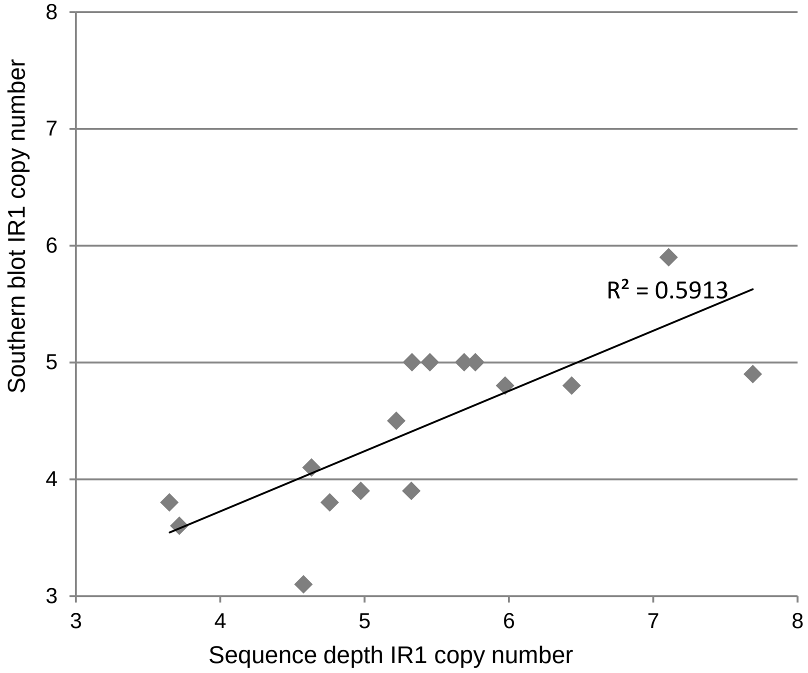

8
7
6
5
4
3
Southern blot IR1 copy number
R² = 0.5913
3
4
5
6
7
8
Sequence depth IR1 copy number

B

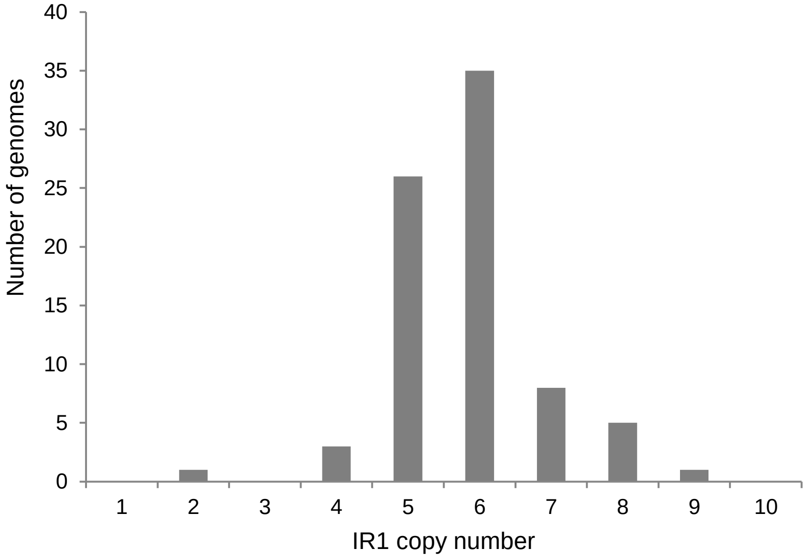

40
35
30
25
20
15
10
5
0
Number of genomes
1
2
3
4
5
6
7
8
9
10
IR1 copy number

Figure A3

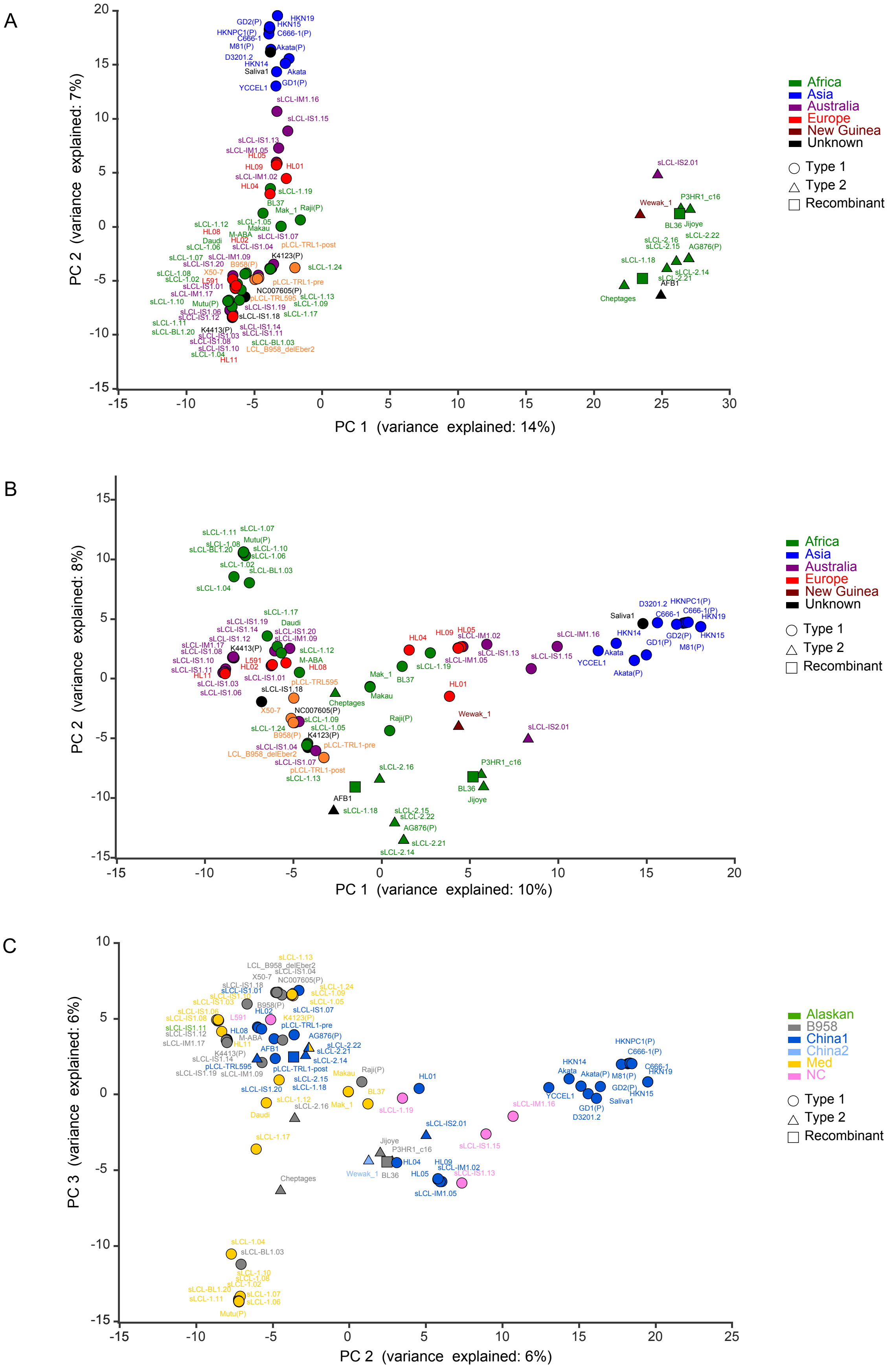
A
PC 1 (variance explained: 14%)
PC 2 (variance explained: 7%)
-15
-10
-5
0
5
10
15
20
25
30
Africa
Asia
Australia
Europe
New Guinea
Unknown
Type 1
Type 2
Recombinant
B
PC 1 (variance explained: 10%)
PC 2 (variance explained: 8%)
Africa
Asia
Australia
Europe
New Guinea
Unknown
Type 1
Type 2
Recombinant
C
PC 2 (variance explained: 6%)
PC 3 (variance explained: 6%)
Alaskan
B958
China1
China2
Med
NC
Type 1
Type 2
Recombinant
